# Olfactory marker protein regulation of glucagon secretion in hyperglycemia

**DOI:** 10.1038/s12276-022-00843-8

**Published:** 2022-09-14

**Authors:** Ju Hun Oh, Ye Eon Han, Ya Ru Bao, Chan Woo Kang, JaeHyung Koo, Cheol Ryong Ku, Yoon Hee Cho, Eun Jig Lee

**Affiliations:** 1 Brain Korea 21 Project for Medical Science, Yonsei University, College of Medicine, Seoul, South Korea; 2 Department of New Biology, DGIST, Daegu, 42988 South Korea; 3 Division of Endocrinology, Department of Internal Medicine, Yonsei University College of Medicine, Seoul, South Korea

**Keywords:** Diabetes insipidus, Cell biology

## Abstract

The olfactory marker protein (OMP), which is also expressed in nonolfactory tissues, plays a role in regulating the kinetics and termination of olfactory transduction. Thus, we hypothesized that OMP may play a similar role in modulating the secretion of hormones involved in Ca^2+^ and cAMP signaling, such as glucagon. In the present study, we confirmed nonolfactory α-cell-specific OMP expression in human and mouse pancreatic islets as well as in the murine α-cell line αTC1.9. Glucagon and OMP expression increased under hyperglycemic conditions. *Omp* knockdown in hyperglycemic αTC1.9 cells using small-interfering RNA (siRNA) reduced the responses to glucagon release and the related signaling pathways compared with the si-negative control. The OMP^lox/lox^;GCG^cre/w^ mice expressed basal glucagon levels similar to those in the wild-type OMP^lox/lox^ mice but showed resistance against streptozotocin-induced hyperglycemia. The ectopic olfactory signaling events in pancreatic α-cells suggest that olfactory receptor pathways could be therapeutic targets for reducing excessive glucagon levels.

## Glucose metabolism: Smell receptor implicated in regulation of blood sugar

The same signaling pathway that controls smell perception in the nose is involved in regulating the release of a pancreatic hormone needed when blood sugar levels are high. The olfactory marker protein (OMP) is a marker of chemical signaling mediated by olfactory receptors, which are located not only in the nose but throughout the body. A research team led by Eun Jig Lee and Yoon Hee Cho from Yonsei University College of Medicine, Seoul, South Korea, showed that human and mouse pancreatic α-cells, endocrine cells that secrete the peptide hormone glucagon to increase glucose levels in the bloodstream, express OMP. Blocking OMPactivity impairs glucagon response pathways. Therapeutics directed at olfactory receptor signaling in the pancreas could therefore help reduce excessive glucagon activity associated with diseases such as type 2 diabetes.

## Introduction

Diabetes mellitus is a chronic metabolic disorder characterized by hyperglycemia. Glucose release into and removal from the circulatory system are important for maintaining normal plasma glucose levels. Insulin is important for controlling plasma glucose levels after meals, as it promotes glucose uptake by peripheral tissues^1^. Glucagon secreted from pancreatic α-cells sustains plasma glucose levels in hypoglycemia by stimulating hepatic glucose production^2^. In type 2 diabetes mellitus (T2DM), insulin resistance and defective insulin release are often accompanied by increased glucagon levels, which further mobilize glucose from the liver^3,4^. Fasting hyperglucagonemia and uncontrolled glucagon levels after meals increase hepatic glucose production and further aggravate diabetes-associated complications. To regulate glucagon secretion, pancreatic α-cells respond appropriately to changes in blood glucose levels (BGLs) by cooperating with both intrinsic and paracrine signals^5,6^. Although a defective glucagon secretory response causes various problems in patients with diabetes, the mechanisms involved in pancreatic α-cell physiology remain elusive, unlike those in β cell physiology, which have been widely studied.

The olfactory marker protein (OMP) is a 19-kDa cytoplasmic protein that is expressed primarily in mature chemosensory neurons in the main olfactory epithelium^7,8^. Recently, OMP has been shown to be expressed in nonolfactory tissues, including those in the endocrine system^9–15^. Previous studies have indicated that OMP acting upstream of cAMP production may be responsible for lowering the elevated Ca^2+^ levels that follow olfactory transduction^16–18^. In pancreatic α-cells, Ca^2+^ and cAMP are key players in the regulation of glucagon secretion^19^. Under physiological conditions, glucagon plays an important role in the counterregulation of glucose, as it is secreted in response to hypoglycemia^20^. Under hypoglycemic conditions, elevated cAMP production promotes glucagon release by increasing intracellular Ca^2+^ influx through the plasma membrane, resulting in secretory granules containing glucagon^21,22^. However, glucagon secretion is inhibited when the glucose concentration is 4–6 mmol/L, which is below the threshold for insulin secretion^23,24^. In contrast, as the glucose concentration increases above 20 mmol/L, causing maximum stimulation of insulin secretion, glucagon secretion is stimulated, resulting in a U-shaped dose–response^2,25^. As OMP contributes to regulating the kinetics and termination of olfactory transduction, we hypothesized that it may play a similar role in modulating the secretion of hormones involved in Ca^2+^ and cAMP signaling.

In this study, we investigated the role of OMP, both in vitro and in vivo, in α-cell models and examined the mechanisms underlying the modulation of glucagon secretion to provide a broader understanding of α-cell physiology. Through immunofluorescence staining of human and rodent pancreases, we confirmed that OMP was selectively expressed in α-cells in pancreatic islets.

The results suggest that OMP contributes to regulating the kinetics of intracellular cAMP production in pancreatic α-cells, which changes the secreted glucagon levels. OMP expression in nonolfactory tissues indicates the existence of potential olfactory receptor (OR)-associated events in nonolfactory systems. Thus, future studies on the regulation of glucagon secretion by targeting ORs expressed on α-cells using odorants will be required for the development of novel treatments for diabetes. This study may provide a rationale for novel treatment regimens for diabetes by targeting OMP or OR signaling to modulate glucagon secretion.

## Materials and methods

### Ethical statement

All animal studies were reviewed and approved by the Institutional Animal Care and Use Committee (IACUC) at Yonsei University Health System (approval number: 2015–0025).

### Human pancreatic tissue samples

The study was approved by the Institutional Review Board at Yonsei University, and written informed consent was obtained from all subjects (IRB number: 4–2013–0299). Pancreatic tissue was obtained from patients with diabetes and nondiabetic patients undergoing pancreatectomy.

### Animal study

Male OMP^−/−^ mice were originally developed by Jackson Laboratory (Bar Harbor, ME, USA) and provided by Prof. Jae Hyung Koo, Department of New Biology, DGIST (Daegu, South Korea). Embryos of the OMP^lox/lox^;GCG^cre/w^ mice were provided by Prof. Pedro Luis Herrera (University of Geneva, Switzerland). All experimental animals were obtained through embryo transfer at our institution and maintained under controlled conditions (12-h light/dark cycle in a temperature- and humidity-controlled environment) with ad libitum access to food and water. For the experiment, 10-week-old mice were starved for 16 h, and 75 mg/kg STZ (Sigma-Aldrich, St. Louis, MO, USA) was intraperitoneally injected daily for 3 consecutive days in the STZ and experimental groups. STZ powder was freshly diluted in 0.1 M sodium citrate buffer (pH 4.5) immediately before injection. The control group was injected with 0.1 M sodium citrate buffer. Body weight and BGL were measured daily before STZ injection and every 2–4 days after the three consecutive STZ injections. Thirteen days after the first STZ injection, the mice were fasted for 16 h, and an oral glucose tolerance test (OGTT) was performed. BGL was measured at 0, 15, 30, 60, and 120 min after the administration of 2 g/kg glucose by the tail nick bleeding method using a glucometer (Arkray, Minneapolis, MN, USA). Blood samples for hormone analysis were collected using the retro-orbital bleeding method, and serum was isolated by centrifuging the blood at 3000 × *g* for 15 min at 4 °C. After OGTT and blood sample collection, the mice were euthanized by CO_2_ inhalation, and pancreatic and liver tissue samples were collected for further analysis. The pancreas of each mouse was fixed in 10% formalin, and paraffin tissue blocks were prepared for staining. The liver tissues were stored in RNAlater solution (Thermo Fisher Scientific, Waltham, MA, USA) at −70 °C until analysis, and RNA and proteins were extracted for the assessment of hepatic enzymes.

### Cell culture

For cell culture, we used a glucagon-secreting mouse αTC1 clone 9 (αTC1.9) cell line donated by Prof. Jae Hyung Koo, Department of New Biology, DGIST (Daegu, South Korea). Cells were cultured in Dulbecco’s modified Eagle’s medium (#31600–034; Thermo Fisher Scientific, Waltham, MA, USA) with 10% (v/v) heat-inactivated FBS, 1% (v/v) penicillin/streptomycin, 2.0 g/L glucose (11.1 mM), 1.5 g/L sodium bicarbonate (17.9 mM), 15 mM HEPES, 0.1 mM nonessential amino acids, and 0.02% (w/v) BSA at 37 °C in a humidified 5% CO_2_ incubator.

### Plasmid constructs and transfection

siRNA targeting mouse *Omp* (5′-AGC AGC UGG CGU GUC AUG AGG UUG G-3′) was purchased from Thermo Fisher Scientific. Before transfection, αTC1.9 cells were seeded at a density of 0.8 × 10^6^ cells/well in 6-well plates for 24 h. siRNA targeting *Omp* or scramble controls (Bioneer, Daejeon, South Korea) was transfected using the Lipofectamine RNAiMAX reagent (Thermo Fisher Scientific) in Opti-MEM medium (Thermo Fisher Scientific) for 16 h. The medium was replaced with culture medium the following day, and the cells were incubated for an additional 24 h before analysis.

### Western blotting

Protein lysates were prepared according to the protein extraction protocol (Cell Signaling Technology, Beverly, MA, USA) with 1 mM phenylmethylsulfonyl fluoride and 1×protease inhibitors (Sigma-Aldrich) and quantified using a bicinchoninic acid protein assay kit (Thermo Fisher Scientific). Thereafter, 30–50 μg of protein lysate was separated by electrophoresis on a 10% SDS-polyacrylamide gel and transferred to polyvinylidene fluoride membranes, which were then blocked with 5% nonfat skim milk and probed with the following primary antibodies at the indicated dilutions: cAMP-responsive element-binding protein (CREB), 1:1000 (#9197); p-CREB, 1:1000 (#9198, all from Cell Signaling Technology, Danvers, MA, USA); and β-actin conjugated horseradish peroxidase (HRP), 1:10000 (47778; Santa Cruz, Paso Robles, CA, USA). The secondary antibody used was donkey anti-rabbit immunoglobulin-horseradish peroxidase antibody (1:5000; Santa Cruz). After incubation with the primary and secondary antibodies, the blots were visualized using a western blotting substrate (WESTSAVEup, AbFrontier, Seoul, South Korea) and exposed to X-ray film (Agfa Healthcare, Mortsel, Belgium). Proteins of interest were quantified using ImageJ v1.51.

### Enzyme-linked immunosorbent assay (ELISA)

αTC1.9 cells were seeded in 6-well plates for 24 h before the experiments. On the day of the experiments, the cells were preincubated in Krebs Ringer bicarbonate (KRB) buffer with 10 mM glucose for 30 min and then incubated with different concentrations of glucose in KRB buffer for 15 min. At the time of harvesting the cells, the media were collected and stored at −70 °C until analysis. The secreted glucagon level was assessed using a glucagon DuoSet ELISA kit (DY1249; R&D Systems, Minneapolis, MN, USA), whereas the circulating insulin levels were measured using an ultrasensitive mouse insulin ELISA kit (90080, Crystal Chem, Elk Grove Village, IL, USA) according to the manufacturers’ instructions.

### RNA isolation, RT-PCR, and quantitative real-time PCR (qRT-PCR)

Total RNA was isolated using NucleoZOL reagent (Macherey-Nagel, Duren, Germany), and cDNA was prepared using ReverTra Ace (TOYOBO, Osaka, Japan) according to the manufacturer’s instructions. The resulting cDNA was subjected to either conventional PCR analysis using Ready-2x-Go [Taq] (Nano Helix, Daejeon, South Korea) or quantitative real-time PCR using the Power SYBR Green PCR Master Mix (Applied Biosystems, Foster City, CA, USA) according to the manufacturers’ instructions.

Information on the primers used in this experiment is reported in Supplementary Table 1.

### Intracellular cAMP measurement

Before the experiments, αTC1.9 cells were seeded in 6-well plates for 24 h. On the day after seeding, the cells were preincubated in KRB buffer with 10 mM glucose. After 30 min, the cells were incubated with a high glucose concentration (30 mM) in KRB buffer for 15 min. The cells were then lysed in 1× cell lysis buffer (Cell Signaling Technology, Beverly, MA, USA) containing 1 mM phenylmethylsulfonyl fluoride dissolved in isopropanol. The protein concentration of 80 μg was determined using the Bradford assay kit (Bio-Rad Laboratories, Hercules, CA, USA), and equal amounts of proteins were loaded. Intracellular cAMP production was assessed using a cyclic AMP assay kit (#4339, Cell Signaling Technology, Beverly, MA, USA) according to the manufacturer’s instructions.

### Cell proliferation assay

Before transfection, αTC1.9 cells were seeded at 0.8 × 10^6^ cells/well in 6-well plates for 24 h. Thereafter, siRNA targeting *Omp* or scramble controls (Bioneer, Daejeon, South, Korea) were transfected using the Lipofectamine RNAiMAX reagent (Thermo Fisher Scientific) in Opti-MEM media (Thermo Fisher Scientific) for 16 h. For the 3-(4,5-dimethylthiazol-2-yl)-5-(3-carboxymethoxyphenyl)-2-(4-sulfophenyl)-2H-etrazolium (MTS) assay, the transfected αTC1.9 cells were replated in 96-well plates (5 × 10^3^ cells/well), and cell survival was assessed at 24, 48, and 72 h after transfection. MTS reagent (Promega, Madison, WI, USA) was diluted in culture medium (10 μL of MTS reagent/100 μL of culture medium), and the cells were incubated at 37 °C for 1 h. Absorbance at 490 nm was recorded using a plate reader (Thermo Fisher Scientific, Waltham, MA, USA).

### Immunofluorescence

Paraffin-embedded samples of the mouse pancreas were cut into 4-µm-thick sections, deparaffinized in xylene, and rehydrated in a graded series of ethanol. After antigen retrieval in 10 mM sodium citrate buffer (pH 6.0), the sections were blocked with 5% normal donkey serum for 1 h and incubated with anti-OMP (1:100, sc-67219; Santa Cruz, CA, USA), anti-glucagon (1:200, ab10988; Abcam, Cambridge, Waltham, MA, USA), and anti-insulin (1:200, ab181547; Abcam) antibodies overnight at 4 °C. After the sections were washed with Tris-buffered saline containing Tween 20, they were incubated with fluorescence-conjugated secondary antibodies, donkey anti-rabbit-FITC (fluorescein isothiocyanate), donkey anti-goat Cy3, or donkey anti-mouse Cy3 (1:200; Jackson ImmunoResearch, West Grove, PA, USA) for 2 h at 20–25 °C. After the sections were washed with PBS containing 0.2% Tween, they were mounted in Vectashield mounting medium with DAPI (Vector Laboratories, Burlingame, CA, USA) and visualized using an LSM 700 laser scanning confocal microscope (Carl Zeiss, Oberkochen, Germany). For colocalization of cells expressing FITC-OMP and Cy3-hormone, ImageJ software was used to calculate Pearson’s coefficient based on the correlation between red and green signal overlap in at least three different pancreatic tissue sections.

αTC1.9 cells were briefly washed with cold PBS, fixed with 4% paraformaldehyde, and then permeabilized with 1% Triton X-100 in PBS. The cells were blocked with 5% normal donkey serum and then incubated with the primary antibodies overnight at 4 °C. Thereafter, the cells were incubated with the secondary antibodies, mounted in Vectashield mounting medium with DAPI, and visualized using an LSM 700 laser scanning confocal microscope (Carl Zeiss).

### Mouse pancreatic islet isolation

Pancreatic islets of 13- to 15-week-old male mice were isolated. Mice were sacrificed with CO_2_ inhalation, and an incision was made immediately around the upper abdomen to expose the liver and intestines. After the ampulla was clamped, 2 mL of liberase solution (1.9 mL RPMI-1640 media + 0.1 mL liberase) was injected into the pancreas through the common bile duct using a 30-gauge needle. The pancreas was carefully removed from the intestine, placed in a 50 mL conical tube containing 2 mL of liberase solution (Roche Holding AG, Basel, Switzerland) and digested for 15 min at 37 °C in a water bath. The conical tube was shaken every 5 min to ensure that the pancreas was completely digested. After complete digestion, 10 mL of Hanks’ balanced salt solution with 0.3% BSA and 1% penicillin/streptomycin was added and filtered through a sieve with a pore size of 200 μm. The islets collected into new conical tubes were centrifuged at 330 × *g* for 2 min, and the supernatant was removed. Islets were resuspended in 10 mL of cold 1100 Histopaque (Sigma-Aldrich, St. Louis, MO, USA), and 10 mL of Hanks’ balanced salt solution was carefully added to the top and then centrifuged at 900 × *g* for 18 min. Isolated islets were poured onto a 100-μm cell strainer, and captured islets were collected into 10-cm culture dishes in RPMI-1640 media with 10% FBS and 1% penicillin/streptomycin. After overnight incubation, islets were hand-picked using a pipette for experiments. Forty islets per sample were used for ELISAs, 150–200 islets per sample were used for western blot analysis, and each experiment was performed in the same way as the αTC1.9 cell line.

### Statistical analysis

Statistical analyses were carried out using GraphPad Prism software v.4.0.0 (GraphPad, Inc., La Jolla, CA, USA). Each experiment was performed at least three times. Statistical significance was determined using one-way ANOVA, followed by post hoc Tukey analysis and Student’s *t*-test to compare the means of two different groups. *p* < 0.05 was considered to indicate significance. Differences are indicated using asterisks (*p < 0.05; **p < 0.01; ***p < 0.001). All measurements were taken from distinct samples.

## Results

### OMP is involved in glucagon secretion by pancreatic α-cells

**Fig. 1:**
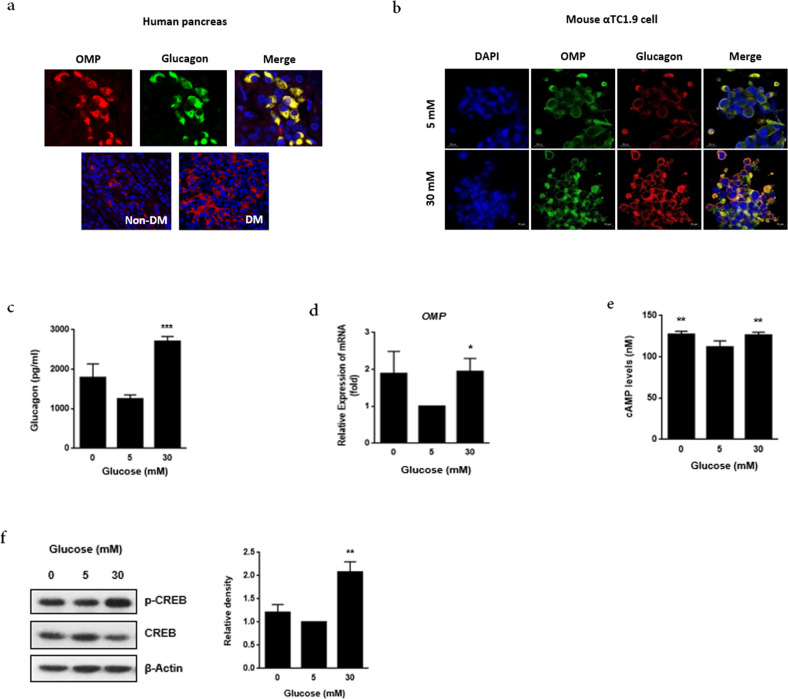
Olfactory marker protein (OMP) is involved in glucose dose–response U-shaped glucagon secretion by pancreatic α-cells. Immunofluorescence staining demonstrated the colocalization of **a** OMP (red) and glucagon (green) in the human pancreas and **b** OMP (green) and glucagon (red) in mouse pancreatic αTC1.9 cells. Images were obtained using a laser scanning microscope at 200x magnification. qRT-PCR analysis of **c** proglucagon *(Gcg)* expression and **d** *Omp* expression in αTC1.9 cells. The mRNA level of each gene was normalized to that of *Gapdh*. Each value is presented as the mean ± SD of three independent experiments. *p < 0.05, **p < 0.01 vs. siNEG, 5 mM glucose, #*p* < 0.05, ##*p* < 0.01 vs. siNEG, 30 mM glucose. **e** Intracellular cAMP levels were analyzed with the indicated glucose concentration for 15 min in αTC1.9 cells. **f** CREB phosphorylation was analyzed using western blotting after 15 min of incubation of cells with the indicated glucose concentrations. Bands are representative of three independent experiments. The two-tailed Student’s *t*-test was used to determine statistical significance. Error bars indicate the standard deviations. *p < 0.05, **p < 0.01, **p < 0.001 vs. 5 mM glucose.

In a previous study, we demonstrated that OMP is expressed only in mouse pancreatic α-cells among the three main types of hormone-producing pancreatic cells^26^. Therefore, we hypothesized that ectopic *Omp*-mediated events would occur in pancreatic α-cells. First, we compared the expression patterns of OMP in human pancreatic islets of nondiabetic individuals and patients with diabetes (Fig. 1a). Consistent with our previous findings, the colocalization of OMP and glucagon was verified in the α-cells of human pancreatic islets.

In the current study, the number of OMP-positive cells was found to be higher in the pancreatic tissues of the patients with diabetes than in those of the nondiabetic individuals. We further examined OMP expression in the glucagon-secreting murine α-cell line αTC1.9 and confirmed the suitability of the cell line for studying the functional role of OMP in α-cells.

In αTC1.9 cells, the colocalization of OMP and glucagon was confirmed, and when the glucose concentration increased from 5 to 30 mM, the expression of OMP and glucagon increased (Fig. 1b). In αTC1.9 cells, glucagon secretion was reduced when the glucose concentration increased from 0 to 5 mM. Conversely, we noted a significant increase in glucagon secretion when the glucose concentration reached 30 mM (*p* < 0.001). Consistent with the findings of previous studies^20,23,27^, we observed a U-shaped dose–response for glucose-regulated glucagon secretion in αTC1.9 cells (Fig. 1c). Interestingly, the *Omp* mRNA levels showed a pattern similar to that of glucose-regulated glucagon secretion (*p* < 0.01 at 30 mM) (Fig. 1d). The changes in intracellular cAMP and p-CREB levels were also consistent with the established pattern of glucagon secretion in response to different glucose concentrations (Fig. 1e, f).

### Glucagon secretion in isolated pancreatic islets of *Omp*^+/+^ and *Omp*^−/−^ mice

**Fig. 2:**
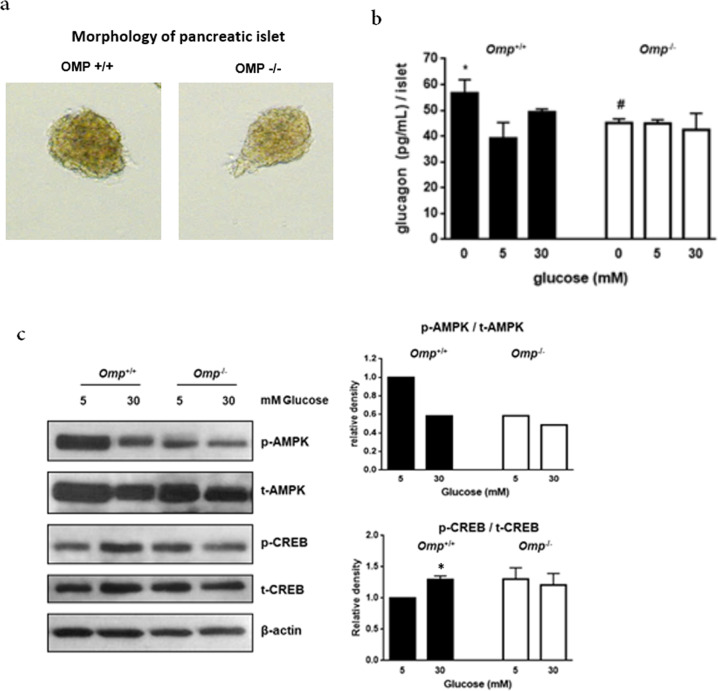
Olfactory marker protein (OMP) is required for glucagon secretion stimulated by high glucose concentrations in isolated mouse pancreatic islets. **a** Morphology of isolated pancreatic islets of the *Omp*^+/+^ and *Omp*^−/−^ mice at 10 weeks. **b** Secreted glucagon levels of isolated pancreatic islets of the *Omp*^+/+^ (*n* = 4) and *Omp*^−/−^ (*n* = 6) mice treated with different concentrations of glucose for 45 min. **c** Phosphorylation of CREB and AMPK was analyzed using western blot analysis after 45 min of incubation with glucose at the indicated concentrations. Phosphorylation of AMPK and CREB was quantified by densitometry and normalized to total AMPK and CREB, respectively, for each sample. The two-tailed Student’s *t*-test was used to determine statistical significance. Error bars indicate the standard deviations. *p < 0.05, **p < 0.01, **p < 0.001 vs. 5 mM glucose, #*p* < 0.05, ##*p* < 0.01 vs. *Omp*^+/+^ at each glucose concentration.

To investigate the role of OMP in glucagon secretion independent of systemic effects, we isolated pancreatic islets of wild-type *Omp*^+/+^ and *Omp*^−/−^ mice. No significant differences were observed between the wild-type *Omp*^+/+^ and *Omp*^−/−^ islets in morphology or size (Fig. 2a). In the wild-type *Omp*^+/+^ islets, the response to different glucose concentrations also showed U-shaped curves similar to those of αTC1.9 cells. However, in the *Omp*^−/−^ islets, the response to both low and high glucose concentrations was blunted. Secretion under 5 mM basal glucose conditions was slightly but not significantly higher than that of the wild-type *Omp*^−/−^ islets (Fig. 2b). This result was in agreement with our in vitro and in vivo experiments. In the *Omp*^−/−^ islets, AMPK phosphorylation was reduced (Fig. 2c). Inhibition of AMPK phosphorylation at 30 mM glucose was not as dramatic in the *Omp*^−/−^ islets as the wild-type *Omp*^+/+^ islets (Fig. 2c). Additionally, phosphorylation of CREB at 5 mM glucose was increased in the *Omp*^−/−^ islets, which is comparable to the 30 mM glucose condition of the *Omp*^+/+^ islets. However, at 30 mM glucose, phosphorylation of CREB was attenuated in the *Omp*^−/−^ islets. These findings suggest that isolated pancreatic islets respond to different glucose concentrations in a dose-dependent U-shaped manner, as in αTC1.9 cells.

### Effect of Omp knockdown on glucose-dependent glucagon secretion in αTC1.9 cells

**Fig. 3:**
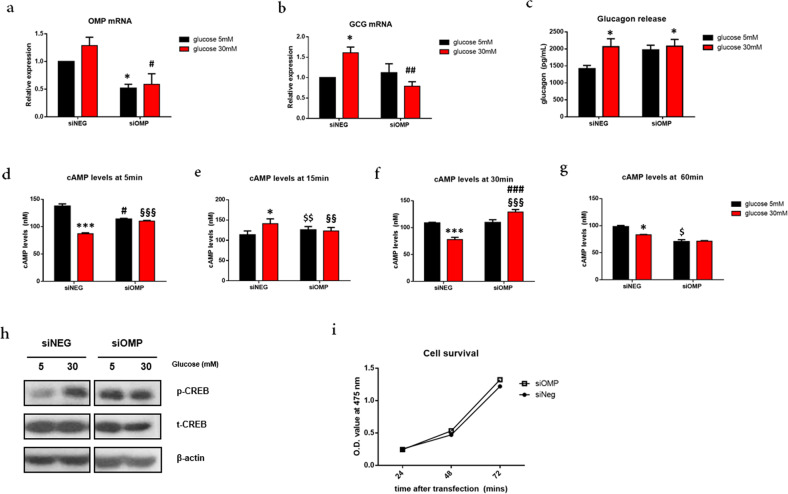
Olfactory marker protein (OMP) knockdown alters the cAMP response, resulting in decreased glucagon secretion at high glucose concentrations. **a**, **b** qRT-PCR analysis of **a** Omp expression and **b** proglucagon *(Gcg)* expression after transfection with *Omp*-specific siRNA in αTC1.9 cells. **c** Analysis of secreted glucagon levels in conditioned medium of transfected αTC1.9 cells with different concentrations of glucose. **d**–**g** Intracellular cAMP levels were analyzed with the indicated glucose concentration for 15 min in αTC1.9 cells transfected with siNEG or siOMP. *p < 0.05, siNEG 5 mM glucose vs. siNEG 30 mM glucose, #*p* < 0.05, siOMP 5 mM glucose vs. siOMP 30 mM glucose, $*p* < 0.05, siNEG 5 mM glucose vs. siOMP 5 mM glucose, §*p* < 0.05, siNEG 30 mM glucose vs. siOMP 30 mM glucose. **h** CREB phosphorylation was analyzed using western blotting after 15 min of incubation of transfected cells with the indicated glucose concentrations. **i** After transfection, αTC1.9 cell survival was assessed using the MTS assay at the indicated time points. The two-tailed Student’s *t*-test was used to determine statistical significance. Error bars indicate the standard deviations. *p < 0.05, vs. 5 mM glucose, #*p* < 0.05, ##*p* < 0.01 vs. siScramble at each glucose concentration.

To determine whether the modulation of OMP expression would affect glucagon secretion and related pathways, we treated αTC1.9 cells with siRNA directed against *Omp* (siOMP) and found that the *Omp* mRNA levels were significantly reduced (*p < 0.05). Under hyperglycemic conditions, the increase in *Omp* expression was significantly higher in the negative control (siNEG) cells than in the OMP knockdown α-cells (##*p* < 0.01) (Fig. 3a). Consistent with these results, the mRNA levels of *Gcg* were increased in the negative control group (*p < 0.05) when the glucose concentration was 30 mM (Fig. 3b). In contrast, 30 mM glucose decreased glucagon expression (##*p* < 0.01 vs. siNEG, Fig. 3c) and CREB phosphorylation (Fig. 3h) in the siOMP-transfected αTC1.9 cells. In the *Omp* knockout animal models, olfactory response kinetics and termination are slowed by 2- to 8-fold due to modulation in cAMP kinetics^17^. Therefore, we investigated whether elevated glucagon secretion is due to altered cAMP kinetics in the siOMP-transfected group of αTC1.9 cells (Fig. 3d–g). For control samples, a rise in cAMP levels occurs at early time points and gradually decreases at 5 mM glucose concentrations, and there is a peak at 15 min at 30 mM glucose. In the siOMP-transfected samples, the kinetics seemed to be delayed, as cAMP levels at 5 min were higher than those at 1 min for 5 mM glucose concentrations, and the peak concentration for the 30 mM glucose concentration was at 30 min. Interestingly, the overall secretions in the siOMP-transfected αTC1.9 cells were elevated compared with those in the control, although stimulation of these cells was attenuated at the high glucose concentration. These results indicate that there is a delay in cAMP responses to glucose when *Omp* is knocked down, which results in elevated glucagon secretion. Cell survival was assessed using the MTS assay to investigate whether glucagon secretion and related pathways were enhanced due to increased cell proliferation. However, we found that *Omp* knockdown had no impact on cell survival (Fig. 3i).

### Glucose homeostasis is altered following STZ induction in the OMP^lox/lox^;GCG^cre/w^ mice

**Fig. 4:**
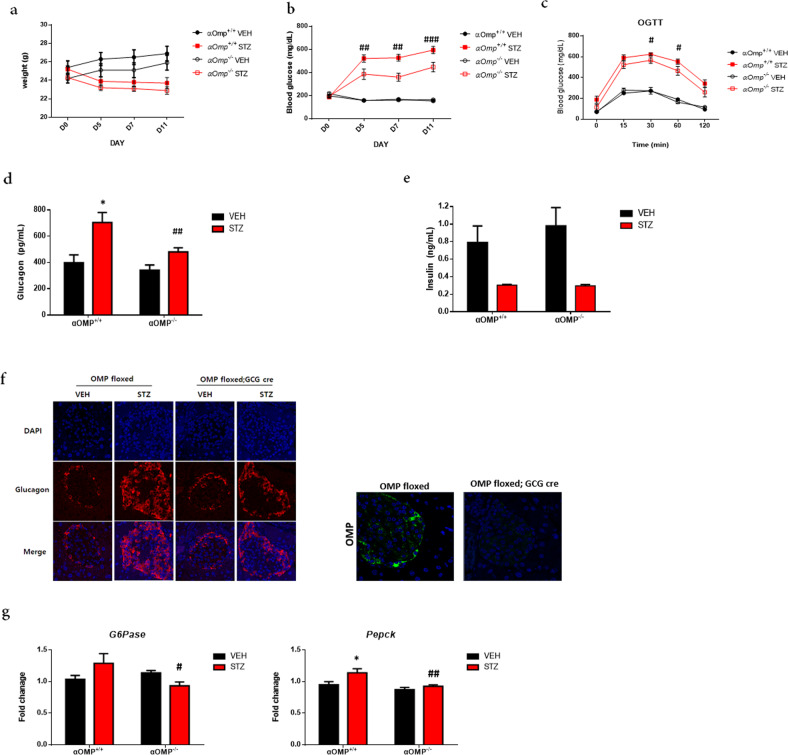
Olfactory marker protein (OMP) is required for high glucose concentration-induced glucagon secretion in α cells of mice. **a** Body weight changes in the OMP^lox/lox^ (αOmp^+/+^) mice and OMP^lox/lox^;GCG^cre/w^ (αOmp^−/−^) mice before and after STZ injection. **b** Blood glucose level changes. ##*p* < 0.01, ###*p* < 0.001 vs. OMP^lox/lox^ STZ. **c** OGTT was performed on Day 13 after STZ injection. On Day 13 after STZ induction, fasting basal glucagon (**d**) and insulin (**e**) levels were measured. **f** Expression of glucagon (red) and OMP (green) in the pancreatic islets of the OMP^lox/lox^;GCG^cre/w^ mice with STZ-induced hyperglycemia. The two-tailed Student’s *t*-test was used to determine statistical significance. Error bars indicate the standard deviations. *p < 0.05 vs. VEH, in each group, #*p* < 0.05, ##*p* < 0.01, ###*p* < 0.001 vs. αOmp^+/+^ STZ. **g** At Day 13 after STZ induction, mice were sacrificed, and liver enzymes were analyzed. The expression of *G6pase* and *Pepck* was analyzed using qRT-PCR analysis in αOmp^+/+^ and αOmp^−/−^. The two-tailed Student’s *t*-test was used to determine statistical significance. Error bars indicate the standard deviations. *p < 0.05 vs. VEH, in each group, #*p* < 0.05, ##*p* < 0.01 vs. αOmp^+/+^ STZ.

Next, we examined whether OMP plays a physiological role in glucagon secretion and glucose homeostasis using the OMP^lox/lox^;GCG^cre/w^ transgenic mouse model. The control (sodium citrate) groups of both wild-type OMP^lox/lox^ mice and OMP^lox/lox^;GCG^cre/w^ mice showed a similar weight range, and weight loss following STZ injection decreased similarly in both the wild-type OMP^lox/lox^ STZ (*p* < 0.005 vs. the wild-type OMP^lox/lox^ control on Day 11) and the OMP^lox/lox^;GCG^cre/w^ STZ groups (*p* < 0.001 vs. the wild-type OMP^lox/lox^ control on Day 11) (Fig. 4a). The feeding BGL of the STZ-injected wild-type OMP^lox/lox^ mice reached 593.7 ± 31.0 mg/dL, whereas that of the OMP^lox/lox^;GCG^cre/w^ mice reached 446.3 ± 40.5 mg/dL on Day 11 (##*p* < 0.01, ###*p* < 0.005 vs. wild-type OMP^lox/lox^ STZ) (Fig. 4b).

OGTT revealed no glycemic excursions in the OMP^lox/lox^;GCG^cre/w^ and wild-type OMP^lox/lox^ mice and that the former was significantly resistant to STZ-induced hyperglycemia (#*p* < 0.05 at 30 and 60 min) (Fig. 4c). We hypothesized that the significant resistance against STZ-induced hyperglycemia in the OMP^lox/lox^;GCG^cre/w^ mice was caused by altered glucagon secretion from OMP-deleted α-cells. As expected, the responses of the OMP^lox/lox^;GCG^cre/w^ mice to oral glucose administration were different from those of the wild-type OMP^lox/lox^ mice. In the fasting state, the basal glucagon levels in the OMP^lox/lox^;GCG^cre/w^ and OMP^lox/lox^ control mice were similar. However, STZ-induced fasting glucagon secretion levels were significantly lower (##*p* < 0.01) in the OMP^lox/lox^;GCG^cre/w^ mice (480.6 ± 31.1 pg/mL) than in the control mice (703.8 ± 76.1 pg/mL) (Fig. 4d). Moreover, STZ injection reduced insulin secretion in both the control and OMP^lox/lox^;GCG^cre/w^ mice (Fig. 4e). As shown in Fig. 4d, the expression of glucagon in pancreatic islets showed a pattern similar to that of glucagon secretion. The expression of glucagon was increased in pancreatic islets of the STZ-treated wild-type OMP^lox/lox^ mice, whereas it was decreased in the OMP^lox/lox^;GCG^cre/w^ mice (Fig. 4f). These findings suggest that *Omp* plays a key role in glucagon secretion changes following STZ induction in the OMP^lox/lox^;GCG^cre/w^ mice.

As the liver is the main target organ for glucagon, where it stimulates hepatic glucose production^28–30^, the expression levels of hepatic enzymes were assessed. As expected, the mRNA levels of enzymes involved in gluconeogenesis, including *glucose 6-phosphatase* (*G6Pase*) (*p* < 0.05) and *phosphoenolpyruvate carboxykinase* (*Pepck*), were upregulated in the STZ-stimulated *Omp*^+/+^ mice and *Omp*^lox/lox^ mice, while STZ-induced glucagon secretion was blunted in the OMP-/-OMP^lox/lox^;GCG^cre/w^ mice, so Pepck and G6pase expression was reduced (*p* < 0.01) (Fig. 4g).

## Discussion

OMP is selectively expressed in the main olfactory epithelium and has also been shown to play roles in nonolfactory tissues, including endocrine tissues^7,8,26,31^. In the olfactory system, the binding of specific ligands to ORs expressed on the surface of OR neurons initiates a signal transduction cascade involving G_olf_, adenylyl cyclase (type III), and OMP, which leads to increased intracellular cAMP production and Ca^2+^ influx. Accumulating evidence indicates that OMP might be important in Ca^2+^ extrusion, acting upstream of cAMP production^17,18,32^.

In pancreatic α-cells, Ca^2+^ and cAMP are key messengers in the regulation of glucagon secretion^33^. Under hypoglycemic conditions, increased cAMP production promotes glucagon release by increasing Ca^2+^ influx, resulting in the exocytotic release of secretory granules containing glucagon^21,22^. Under normal physiological conditions, glucose inhibits glucagon secretion by lowering the Ca^2+^ concentration in a cell. However, when the glucose concentration exceeds a specific threshold, above 25 mmol/l, glucose stimulates the release of glucagon independent of the Ca^2+^ concentration. Instead, glucagon secretion is further promoted by cAMP^27^. As OMP plays a role in regulating the kinetics and termination of olfactory transduction involving Ca^2+^ and cAMP signaling, we hypothesized that OMP may play a similar role in modulating glucagon secretion in pancreatic α-cells.

In a previous study, immunofluorescence staining for OMP and the three main pancreatic hormones, glucagon, somatostatin, and insulin, to determine the expression of OMP in pancreatic islets of mice revealed that OMP is specifically expressed in glucagon-positive α-cells; immunofluorescence staining of the human pancreas showed similar results^26^. After confirming the expression of OMP in the glucagon-secreting murine α-cell line αTC1.9, we investigated whether the modulation of OMP expression would affect glucose-regulated glucagon secretion and related signaling pathways. Interestingly, *Omp* knockdown in αTC1.9 cells increased basal glucagon secretion as well as intracellular cAMP levels and CREB phosphorylation.

In the olfactory system, the absence of OMP results in delayed time-to-transient-peak of response, latency to first spike, and response termination. These phenomena have been demonstrated to be caused by the increased overall basal activity due to the prolonged response of cAMP in *Omp*^−/−^ mice in response to odorants^17^. Recent studies have reported that OMP directly binds cAMP to regulate the level of freely available cAMP and to control the basal cAMP pool^34–36^. Thus, by buffering the cAMP levels, OMP prevents persistent depolarization of OR neurons, enabling a sustained response to repeated stimuli. We predicted that, like α-cells, OMP would bind newly generated cAMP to prevent excessive surge and enable glucagon secretion. As expected, delayed intracellular cAMP kinetics were observed when *Omp* was knocked down, resulting in elevated overall basal glucagon secretion. Interestingly, in the siOMP-transfected α-cells, stimulation by low or high glucose concentrations was attenuated. Increased basal activity and prolonged response of cAMP in the absence of OMP indicate that OMP works upstream of cAMP production. Moreover, *Omp* knockdown resulted in weak cAMP buffering and reduced glucagon secretion due to sustained depolarization after the initial cAMP surge. OMP knockdown does not affect cell proliferation; therefore, glucagon secretion changes are independent of α-cell proliferation.

As in the in vitro experiments, circulating glucagon levels in the control groups were not significantly different between the wild-type OMP^lox/lox^ and OMP^lox/lox^;GCG^cre/w^ mice under fasting basal conditions. However, the increase in glucagon release due to STZ-induced hyperglycemia was significantly lower in the OMP^lox/lox^;GCG^cre/w^ mice than in the wild-type OMP^lox/lox^ mice. Consistent with this result, OGTT demonstrated that the OMP^lox/lox^;GCG^cre/w^ mice had significantly fewer glycemic excursions than the wild-type *Omp*^+/+^ mice. As there were no significant changes in the plasma levels of insulin and GLP-1, which are important hormones controlling BGL, the altered glucose homeostasis in the OMP^lox/lox^;GCG^cre/w^ mice may arise due to changes in glucagon secretion.

Moreover, the expression of enzymes in the liver, which is the main target organ of glucagon, was affected by altered glucagon release^30^. Under normal physiological conditions, glucagon stimulates the export of glucose from the liver by increasing the rates of glycogenolysis and gluconeogenesis and suppressing glycolysis^37,38^. As the basal circulating glucagon levels decreased in the OMP^lox/lox^;GCG^cre/w^ mice, the mRNA levels of enzymes involved in gluconeogenesis, including *G-6pase* (*p* < 0.01) and *Pepck*, were decreased in STZ-induced hyperglycemia. Consequently, the STZ-injected OMP^lox/lox^;GCG^cre/w^ mice were more resistant to hyperglycemia than the OMP^lox/lox^ mice.

Nevertheless, there are certain limitations to this study. We did not determine whether the changes in glucagon secretion due to OMP are limited to STZ-induced hyperglycemia alone or diabetes in general, such as high-fat diet-induced hyperglycemia. However, we used STZ-injected mice because we excluded factors that regulate glucagon secretion except for OMP. STZ damages pancreatic β-cells, which secrete insulin, thus inhibiting glucagon secretion and inducing hyperglycemia. Therefore, the STZ-induced diabetic mouse model was more suitable to study the relationship between OMP and glucagon secretion.

In conclusion, OMP is responsible for regulating the cAMP kinetics of glucagon secretion in α-cells of the pancreatic islet. In vitro studies using αTC1.9 cells revealed that delayed responses in cAMP kinetics to different glucose concentrations result in elevated glucagon secretion when OMP is absent. The basal secretion of glucagon was already elevated; therefore, stimulatory effects at low and high glucose concentrations were attenuated. Notably, the fasting circulating glucagon levels in the OMP^lox/lox^;GCG^cre/w^ mice were similar to those in the wild-type OMP^lox/lox^ mice, but the degree of hyperglucagonemia induced by STZ was significantly reduced in the former, resulting in significant resistance against STZ-induced hyperglycemia.

Our findings provide evidence that OMP expression in α-cells has a physiological role in regulating glucagon secretion, especially in hyperglycemia. The finding of ectopic olfactory signaling events in pancreatic α-cells suggests that OR pathways could be targets for reducing glucagon levels in patients with diabetes. Inhibiting olfactory signaling in diabetic α-cells could allow the development of novel treatment regimens for diabetes.

## Supplementary information

Gene, primer orientation, primer sequence (5′ to 3′), and sequence for primers used in real-time quantitative PCR assays
