# Supplementary material for: Olfactory marker protein regulation of glucagon secretion in hyperglycemia
Source: Exp Mol Med. 2022 Sep 14;54(9):1502–10. doi: 10.1038/s12276-022-00843-8 (PMC9534918; doi:10.1038/s12276-022-00843-8)
Supplement: Supplementary file 1 — Gene, primer orientation, primer sequence (5′ to 3′), and sequence for primers used in real-time quantitative PCR assays [file 12276_2022_843_MOESM1_ESM.pdf]

Supplementary Table 1

Gene (mouse)	Primer Sequence	Product Size (bp)	Tm (℃)
Gapdh	Forward: AACTTTGGCATTGTGGAAGG	223	56.75
	Reverse: ACACATTGGGGGTAGGAACA		58.55
Gcg	Forward: ACTCACAGGGCACATTCACC	111	60.25
	Reverse: TGGCAATGTTGTTCCGGTTC		59.33
Omp	Forward: AGTGTCCCCTGACTGTCTCA	139	59.81
	Reverse: ACCGGTACCACAGCCTATCT		60.03
G6pase	Forward: CTGTTTGGACAACGCCCGTAT	91	61.21
	Reverse: AGGTGACAGGGAACTGCTTTA		58.95
Pepck	Forward: CTGCATAACGGTCTGGACTTC	21	58.73
	Reverse: GCCTTCCACGAACTTCCTCAC		61.21

Supplementary Table 1. Gene, primer orientation, primer sequence (5′ to 3′), and sequence for primers used in real-time quantitative PCR assays
